# Association between sensitivity to thyroid hormone and prognosis in septic patients: a retrospective cohort analysis

**DOI:** 10.3389/fendo.2025.1611963

**Published:** 2025-08-27

**Authors:** Jia Wang, Yingbai Wang, Zihan Xu, Chuxun Chou, Jiaofeng Xiang, Xizhi Zhang, Xuefei Hou, Suru Yue, Shicai Ye, Feng Chen, Jiayuan Wu

**Affiliations:** ^1^ Clinical Research Service Center, Affiliated Hospital of Guangdong Medical University, Zhanjiang, Guangdong, China; ^2^ Guangdong Engineering Research Center of Collaborative Innovation of Clinical Medical Big Data Cloud Service in Western Guangdong Medical Union, Affiliated Hospital of Guangdong Medical University, Zhanjiang, Guangdong, China; ^3^ Department of Gastroenterology, Affiliated Hospital of Guangdong Medical University, Zhanjiang, Guangdong, China; ^4^ Department of Critical Care Medicine, Affiliated Hospital of Guangdong Medical University, Zhanjiang, Guangdong, China

**Keywords:** sepsis, thyroid hormones, thyroid hormone sensitivity indices, Cox proportional hazards model, K-means clustering analysis

## Abstract

**Purpose:**

Sepsis is associated with significant endocrine dysfunction, particularly in thyroid hormone metabolism. This study aims to investigate the association between thyroid hormone sensitivity indices and prognosis in sepsis, exploring their potential as early prognostic markers.

**Methods:**

We conducted a retrospective analysis of sepsis patients admitted to the Affiliated Hospital of Guangdong Medical University. Nonlinear associations between thyroid hormones (FT3, FT4, TSH), sensitivity indices (FT3/FT4, TFQI, PTFQI, TSHI, TT4RI), and sepsis mortality were assessed using restricted cubic spline models. Kaplan-Meier curves along with Cox proportional hazards models were used to investigate the longitudinal associations. K-means clustering was applied to thyroid hormone profiles to identify distinct phenotypes.

**Results:**

Among 2,391 sepsis patients, non-survivors exhibited significantly lower levels of thyroid hormone and sensitivity indices compared to survivors. Restricted cubic spline analysis revealed a nonlinear dose-response relationship, with lower FT3, TFQI, PTFQI, TSHI, and TT4RI levels associated with increased mortality risk. Multiple Cox regression models identified FT3 (HR = 0.95, 95% CI: 0.93–0.98, *p* = 0.001), TSH (HR = 0.89, 95% CI: 0.80–0.99, *p* = 0.004), TFQI (HR = 0.66, 95% CI: 0.51–0.84, *p* < 0.001), PTFQI (HR = 0.47, 95% CI: 0.37–0.61, *p* < 0.001), TSHI (HR = 0.92, 95% CI: 0.85–0.99, *p* = 0.040), and TT4RI (HR = 0.98, 95% CI: 0.97–0.99, *p* = 0.001) as independent predictors of 90-day mortality. K-means clustering identified two distinct phenotypes, with Phenotype 2, characterized by profound thyroid hormone suppression and reduced sensitivity indices, was associated with a 36% higher mortality risk (HR = 1.42, 95% CI: 1.04–1.91, *p* = 0.029).

**Conclusion:**

Impaired thyroid hormone sensitivity are significantly associated with increased mortality in sepsis, emphasizing their potential as prognostic biomarkers and suggest their utility in risk stratification and personalized management of sepsis patients.

## Introduction

Sepsis, a life-threatening condition triggered by acute infection, is characterized by a dysregulated host response and multiple-organ dysfunction, contributing significantly to preventable mortality among critically ill patients (1). Despite advances in diagnostics and therapeutics, sepsis remains a global health crisis, with 48.9 million incident cases and a mortality rate of 22.5% worldwide in 2017, accounting for 20% of global deaths (2). Extensive research has identified biomarkers such as lipoproteins (3), interleukins (4), and heparin-binding protein (HBP) (5) in sepsis. However, sepsis is fundamentally a systemic endocrine disorder, with dysregulation of the neuroendocrine-immune network playing a critical role in disease progression and outcomes (6). Among these endocrine disturbances, thyroid hormonal dysfunction is particularly prevalent and may serve as a key determinant of clinical prognosis.

Thyroid hormones are central to metabolic homeostasis, especially under stress and critical illness. In hospitalized patients, especially older individuals or those with severe conditions, fluctuations in thyroid hormone levels are common (7). These changes, often transient and not indicative of intrinsic thyroid disease (8), including reductions in serum triiodothyronine (T3) and free triiodothyronine (FT3) concentrations in both acute and chronic critical illness (9). Prolonged critical illness may further lead to declines in thyroxine (T4) and thyroid-stimulating hormone (TSH), a condition termed “nonthyroidal illness syndrome” (NTIS) (10). Since its identification in the 1970s, NTIS has been recognized as a predictor of poor prognosis in critically ill patients (11), complicating a wide range of critical illnesses across all age groups, from preterm infants to adults (12). In the Intensive Care Unit (ICU), NTIS is associated with adverse disease trajectories and increased mortality (13, 14).

Traditional thyroid function assessments, however, have significant limitations. Isolated measurements of T3, T4, and TSH fail to fully capture thyroid hormone homeostasis (15), and conventional reference ranges often overlook the influence of age and sex. For instance, age- and sex-adjusted reference ranges have revealed that up to 40% of subclinical hypothyroidism cases are misdiagnosed (16). While current reference ranges aid in diagnosing thyroid dysfunction, their relevance to adverse outcomes in critical diseases remains unclear (17).

To address these limitations, researchers have developed indices to assess impaired thyroid hormone sensitivity. Peripheral sensitivity indices, such as the FT3/FT4 ratio (18), and central sensitivity indices, including the thyroid feedback quantile-based index (TFQI) (19), parametric thyroid feedback quantile-based index (PTFQI) (19), thyroid-stimulating hormone index (TSHI) (20), and thyrotrophic thyroxine resistance index (TT4RI) (21), have been proposed. Recent researches have re-evaluated thyroid function in various diseases through these indices, suggesting that they may better capture phenotypic heterogeneity and offer superior prognostic value compared to traditional thyroid hormone parameters. Impaired thyroid hormone sensitivity has been linked to cardiovascular disease (22), hypertension (23), non-alcoholic fatty liver disease (24), and obesity (22). Despite these advances, no studies have explicitly investigated the association between thyroid hormone sensitivity and sepsis prognosis. While altered thyroid hormone levels are associated with adverse outcomes in sepsis, the role of thyroid hormone sensitivity as an independent prognostic indicator remains underexplored.

This study aims to retrospectively analyze early thyroid hormone levels in sepsis patients, focusing on the predictive value of thyroid hormone sensitivity indices for in-hospital prognosis in septic patients. By addressing this critical knowledge gap, we seek to provide a theoretical foundation for targeted interventions and improved patient outcomes.

## Methods

### Study population and design

This retrospective cohort study analyzed data from sepsis patients admitted to the ICU of the Affiliated Hospital of Guangdong Medical University between January 2013 and July 2024. Patients were included if they met the Sepsis 3.0 diagnostic criteria (1), were age ≥ 18 years, and had complete clinical data. Exclusion criteria included: (1) > 25% missing clinical data (N = 789), (2) pre-existing thyroid diseases or autoimmune disorders (N = 92), (3) hypothalamic-pituitary diseases or endocrine/metabolic disorders (N = 26), and (4) use of medications known to interfere with thyroid function, such as glucocorticoids, dopamine, or norepinephrine (N = 166). For patients with multiple admissions, only the first hospital admission was included. The study adhered to the Declaration of Helsinki and received ethical approval from the Ethics Committee of the Affiliated Hospital of Guangdong Medical University (Approval number: PJKT2024-255). Reporting followed the Strengthening the Reporting of Observational Studies in Epidemiology (STROBE) guidelines (25). All participants in this study provided written informed consent. This study did not include any minor participants.

### Assessment of thyroid hormone sensitivity

Thyroid hormone parameters, including free triiodothyronine (FT3), free thyroxine (FT4), and thyroid-stimulating hormone (TSH), were measured on the first day of hospitalization using electrochemiluminescence immunoassay (ECLIA) on an automated biochemical analyzer. Reference ranges were as follows: for FT3, 3.9–6.0 pmol/L; FT4, 12.2–20.1 pmol/L; and TSH, 0.71–4.92 mIU/L (26). The FT3/FT4 ratio, reflecting peripheral thyroid hormone conversion efficiency, was calculated by FT3 divided by FT4 with lower values indicating reduced conversion (10). This ratio directly reflects the efficiency of peripheral tissues in converting the thyroid hormone prohormone (T4) into its active form (T3).

Central thyroid hormone sensitivity was assessed using the thyroid feedback quantile-based index (TFQI) and its parametric counterpart (PTFQI). TFQI is a composite index constructed based on reference percentiles from a healthy population. It quantifies the deviation between the observed TSH level and its expected position within the healthy population at a given FT4 level, which was calculated as TFQI = cdfFT4 - (1 - cdfTSH) (19). PTFQI is a variant of TFQI, primarily designed to identify and quantify central thyroid hormone resistance (positive PTFQI values indicate greater resistance). It was derived using the formula PTFQI = Φ((FT4 – *μ*FT4)/*σ*FT4) − (1 − Φ((ln TSH − *μ*lnTSH)/*σ*lnTSH)) (21), where *μ* FT4 = 16.3802, *σ* FT4 = 1.98049, *μ*lnTSH = 0.5865, and *σ*lnTSH = 0.43854 for the Chinese population (27). Both TFQI and PTFQI range from -1 to 1, with negative values indicating preserved sensitivity and positive values reflecting impaired sensitivity. Additional indices included the thyroid-stimulating hormone index (TSHI), which was calculated as TSHI = ln(TSH)+0.1345×FT4, and the thyrotroph thyroxine resistance index (TT4RI), which was calculated as TT4RI = FT4×TSH. TSHI aims to normalize the inherently right-skewed distribution of raw TSH values (characterized by most values concentrated at the lower end with a long tail of extreme high values) through mathematical transformation, thereby improving its approximation to a normal distribution and linearizing its relationship with FT4. TT4RI quantifies the difference between the actual FT4 level and the model-predicted “ideal” FT4 level at a given TSH concentration.

### Outcome

The primary outcome was the 90-day mortality following septic diagnosis. Follow-up began at diagnosis and ended at either in-hospital death or discharge.

### Covariates

The following covariates were included in the analysis: (1) demographic characteristics, including age, gender, marital status, smoking status, and alcohol consumption; (2) comorbidities, including hypertension, coronary heart disease, diabetes, hepatitis, and infectious diseases; (3) disease severity scores, including Glasgow Coma Scale (GCS) and Acute Physiology and Chronic Health Evaluation (APACHE II); (4) laboratory parameters, including red blood cell count (RBC), white blood cell count (WBC), lymphocyte count, hemoglobin, platelets, mean platelet volume, platelet distribution width, alanine aminotransferase (ALT), aspartate transaminase (AST), total bilirubin, albumin, globulin, total protein, serum creatinine, total cholesterol, potassium, sodium, chloride, calcium, carbon dioxide, anion gap, and glucose; (5) clinical interventions, including surgical procedures and mechanical ventilation.

### Statistical analysis

Continuous variables were expressed as mean ± standard deviation (SD) or median (interquartile range [IQR]), depending on their distribution. Categorical variables were reported as frequencies and percentages (n (%)). Group comparisons used the Wilcoxon rank-sum test for continuous variables and the chi-squared test or Fisher’s exact test for categorical variables.

Nonlinear associations between thyroid hormones (FT3, FT4, and TSH) and sensitivity indices (FT3/FT4, TFQI, PTFQI, TSHI, and TT4RI) with sepsis-related mortality were evaluated using restricted cubic spline models. Continuous variables were categorized into quartiles, and Kaplan-Meier curves with log-rank tests assessed survival differences. Cox proportional-hazard regression models assessed associations between thyroid hormone parameters and 90-day mortality with hazard ratios (HR) and 95% confidence intervals (CI) reported. Three models were constructed through adjusting for various covariates. Model 1 represented a univariate analysis without adjustment for covariates. Model 2 was adjusted for gender, age, marital status, hypertension, coronary artery disease, diabetes, hepatitis, surgery, and mechanical ventilation. Model 3 was further adjusted for GCS, APACHE II, RBC, WBC, hemoglobin, ALT, serum creatinine, potassium, sodium, chloride, calcium, carbon dioxide, anion gap, and glucose, in addition to the covariates in Model 2.

K-means clustering identified patient subgroups based on thyroid hormone profiles using eight indicators: FT3, FT4, TSH, FT3/FT4, TFQI, PTFQI, TSHI, and TT4RI. The optimal number of clusters was identified using the total within-cluster sum of squares (WSS), silhouette score (ranging from -1 to 1, with higher values indicating better clustering), Davies-Bouldin score (ranging from 0 upwards, with lower values indicating better clustering), and Calinski-Harabasz score (ranging from 0 upwards, with higher values indicating better clustering). Clustering results were visualized using principal component analysis (PCA). Kaplan-Meier curves and Cox proportional hazards models validated the prognostic significance of the identified clusters. All statistical analyses were conducted using R software (version 4.3.1), with a two-sided *p*-value < 0.05 considered statistically significant.

## Results

### Characteristics of the study population

Of 3,464 sepsis patients initially screened, 2,391 patients met inclusion criteria and were included in the analysis (**Supplementary Figure S1**). The cohort had a median age of 55 years, with a male predominance (57.76%). Non-survivors were significantly older, had a higher prevalence of comorbidities, and exhibited higher APACHE II scores compared to survivors (all *p* < 0.05, **Table 1**).

**Table 1:** Baseline characteristics of participants with sepsis.

Characteristics	Overall	Survivors	Nonsurvivors	p value
N (%)	2391	2018	373	
Age, year	55.00 [39.00, 69.00]	54.00 [38.00, 68.00]	63.00 [44.00, 75.00]	< 0.001
Gender, n (%)				< 0.001
Male	1381 (57.76)	1135 (56.24)	246 (65.95)	
Female	1010 (42.24)	883 (43.76)	127 (34.05)	
Marital status, n (%)				< 0.001
Married	1750 (73.19)	1451 (71.91)	299 (80.16)	
Single/Divorced	183 (7.65)	151 (7.48)	32 (8.58)	
Unknown	458 (19.16)	416 (20.61)	42 (11.26)	
Admission type, n (%)				< 0.001
Department of Emergency Medicine	411 (17.19)	319 (15.81)	92 (24.66)	
Department of Critical Care Medicine	669 (27.98)	544 (26.96)	125 (33.52)	
Department of Infectious Diseases	761 (31.83)	698 (34.58)	63 (16.89)	
Department of Geriatric Medicine	550 (23.00)	457 (22.65)	93 (24.93)	
Comorbidity, n (%)				
Hypertension	516 (21.58)	398 (19.72)	118 (31.64)	< 0.001
Coronary heart disease	127 (5.31)	97 (4.81)	30 (8.04)	< 0.001
Diabetes	287 (12.00)	228 (11.30)	59 (15.82)	< 0.001
Hepatitis	80 (3.35)	66 (3.27)	14 (3.75)	< 0.001
Infectious disease	153 (6.40)	125 (6.19)	28 (7.51)	< 0.001
Smoking, n (%)	267 (11.17)	220 (10.90)	47 (12.60)	< 0.001
Drinking, n (%)	177 (7.40)	144 (7.14)	33 (8.85)	< 0.001
Surgery, n (%)	1023 (42.79)	793 (39.30)	230 (61.66)	< 0.001
Mechanical ventilation				
Invasive ventilation, n (%)	158 (6.60)	59 (2.92)	99 (26.54)	< 0.001
Non-invasive ventilation, n (%)	230 (9.61)	180 (8.91)	50 (13.40)	< 0.001
None	2003 (83.79)	1779 (88.17)	224 (60.06)	< 0.001
GCS score	14.00 [13.00, 15.00]	15.00 [14.00, 15.00]	12.00 [5.00, 14.00]	< 0.001
APACHE II score	13.00 [10.00, 18.00]	13.00 [9.00, 17.00]	19.00 [14.00, 27.00]	< 0.001
Laboratory				
RBC (10^12/L)	3.89 [3.16, 4.45]	3.88 [3.19, 4.47]	3.93 [3.03, 4.41]	< 0.001
WBC (10^9/L)	9.41 [6.73, 13.49]	9.18 [6.69, 13.1]	10.70 [7.26, 15.57]	< 0.001
Lymphocyte (10^9/L)	1.24 [0.77, 1.78]	1.28 [0.81, 1.81]	1.00 [0.60, 1.55]	< 0.001
Hemoglobin (g/dL)	109.00 [70.00, 128.00]	108.65 [67.88, 128.00]	112.00 [77.00, 130.00]	< 0.001
Platelets (10^9/L)	204.80 [146.00, 264.80]	205.57 [149.00, 266.00]	197.00 [127.40, 259.35]	< 0.001
Mean platelet volume (fL)	9.50 [8.68, 10.32]	9.50 [8.70, 10.30]	9.60 [8.60, 10.60]	< 0.001
Platelet distribution width (fL)	14.00 [11.10, 16.30]	13.80 [11.00, 16.30]	15.40 [11.92, 16.80]	< 0.001
Alanine aminotransferase (U/L)	29.72 [17.12, 47.48]	29.50 [16.90, 47.52]	30.72 [18.26, 47.179]	< 0.001
Aspartate transaminase (U/L)	32.38 [20.70, 48.04]	31.49 [20.32, 47.40]	35.58 [23.60, 51.26]	< 0.001
Total bilirubin (μmol/L)	11.98 [8.58, 18.30]	11.60 [8.38, 17.51]	13.62 [10.01, 21.83]	< 0.001
Globulin (g/L)	29.10 [26.56, 31.60]	29.10 [26.60, 31.67]	28.92 [26.50, 31.32]	< 0.001
Total protein (g/L)	63.68 [59.40, 67.68]	63.83 [59.50, 67.81]	63.08 [59.25, 67.20]	< 0.001
Serum creatinine (mg/dL)	86.00 [65.00, 137.00]	84.00 [64.00, 130.18]	100.92 [69.00, 166.90]	< 0.001
Total cholesterol (mmol/L)	3.91 [3.22, 4.66]	3.90 [3.24, 4.66]	3.91 [3.14, 4.66]	< 0.001
Potassium (mmol/L)	3.81 [3.47, 4.17]	3.81 [3.48, 4.17]	3.78 [3.44, 4.22]	< 0.001
Sodium (mmol/L)	138.20 [135.30, 140.86]	138.20 [135.38, 140.84]	138.50 [135.00, 141.05]	< 0.001
Chloride (mmol/L)	101.62 [98.48, 104.60]	101.70 [98.40, 104.63]	101.60 [98.56, 104.35]	< 0.001
Calcium (mmol/L)	2.11 [1.99, 2.21]	2.11 [2.00, 2.21]	2.09 [1.97, 2.21]	< 0.001
Carbon dioxide (mmol/L)	21.52 [19.21, 23.6]	21.66 [19.30, 23.70]	21.13 [18.92, 23.29]	< 0.001
Anion gap (mmol/L)	14.81 [12.50, 17.34]	14.79 [12.50, 17.30]	15.30 [12.50, 17.84]	< 0.001
Glucose (mmol/L)	6.19 [4.97, 8.12]	6.09 [4.89, 8.02]	7.09 [5.56, 8.90]	< 0.001
Albumin (g/dL)	34.54 [30.80, 36.68]	34.67 [30.86, 38.75]	34.08 [30.37, 38.01]	< 0.001
C-reactive protein (mg/L)	77.80 [30.90, 126.00]	77.20 [30.10, 122.00]	79.90 [35.17, 139.13]	< 0.001
Procalcitonin (ng/mL)	1.06 [0.21, 6.21]	0.95 [0.20, 6.07]	1.60 [0.35, 6.22]	< 0.001
FT3 (pmol/L)	3.13 [2.37, 3.97]	3.21 [2.45, 4.01]	2.82 [2.04, 3.61]	< 0.001
FT4 (pmol/L)	14.90 [12.27, 17.60]	14.92 [12.41, 17.65]	14.32 [11.40, 17.40]	< 0.001
TSH (mIU/L)	1.21 [0.62, 2.16]	1.274 [0.66, 2.19]	0.95 [0.43, 1.76]	< 0.001
FT3/FT4	0.21 [0.16, 0.26]	0.21 [0.17, 0.26]	0.19 [0.14, 0.25]	< 0.001
TFQI	0.02 [-0.30, 0.31]	0.04 [-0.26, 0.33]	-0.07 [-0.42, 0.19]	< 0.001
PTFQI	0.25 [0.01, 0.55]	0.27 [0.03, 0.57]	0.12 [-0.07, 0.44]	< 0.001
TSHI	3.39 [2.59, 4.34]	3.45[2.64, 4.40]	3.06 [2.32, 3.97]	< 0.001
TT4RI	18.18 [8.39, 32.41]	19.11 [9.15, 33.59]	13.19 [4.87, 26.39]	< 0.001

Data are presented as median (interquartile) or number (proportion, %).

RBC, red blood cell; WBC, white blood cell; GCS, Glasgow Coma Scale; APACHE, Acute Physiology and Chronic Health Evaluation; FT3, free triiodothyronine; FT4, free thyroxine; TSH, thyroid-stimulating hormone; TFQI, thyroid feedback quantile-based index; PTFQI, parametric thyroid feedback quantile-based index; TSHI, TSH index; TT4RI, thyrotropin thyroxine resistance index.

Baseline levels of FT3, FT4, and TSH were significantly lower in non-survivors than in survivors (all *p* < 0.05). Similarly, thyroid hormone sensitivity indices, including FT3/FT4, TFQI, PTFQI, TT4RI, and TSHI, were significantly reduced in non-survivors (all *p* < 0.001).

### Restricted cubic spline models

**Figure 1:**
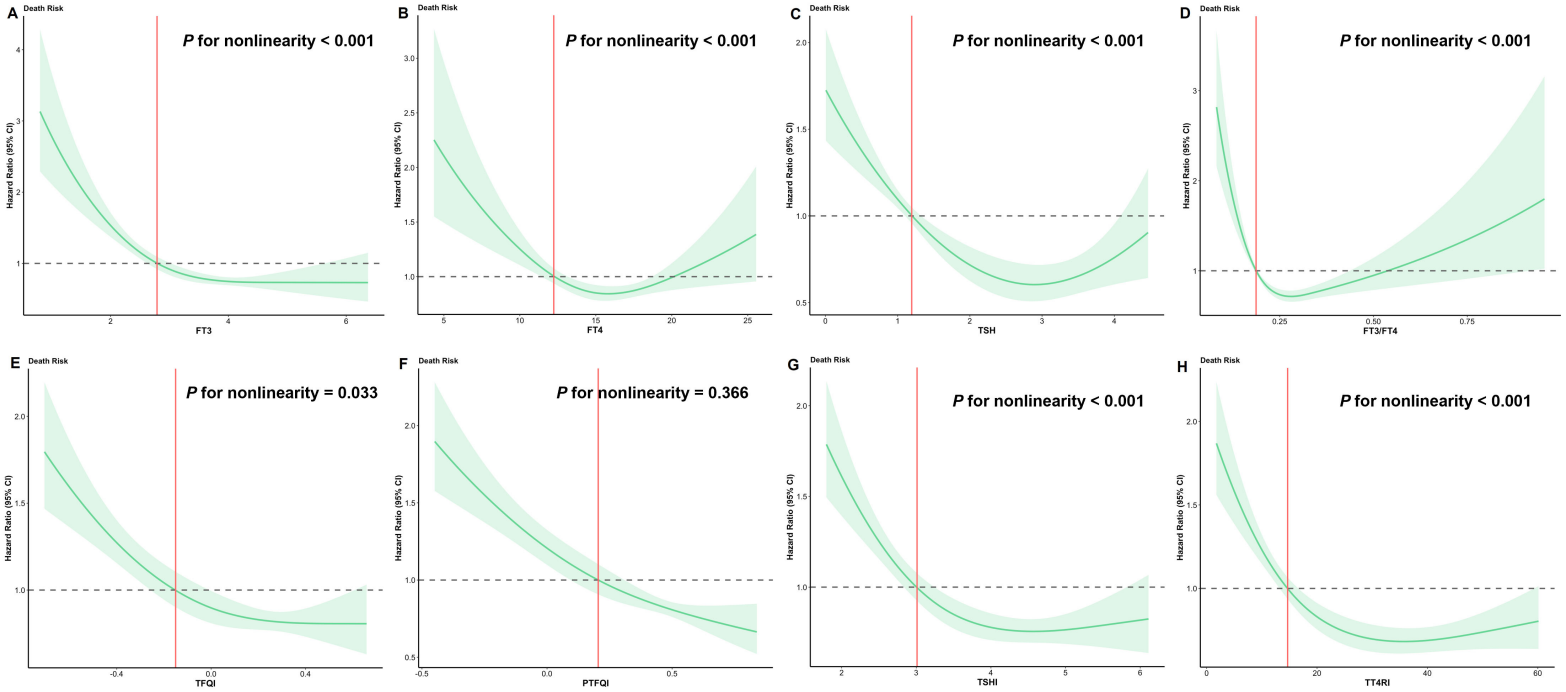
Restricted cubic spline analysis. **(A)** restricted cubic spline models for the relationship between FT3 predicted with the risk of prognosis in patients with sepsis, **(B)** restricted cubic spline models for the relationship between FT4 predicted with the risk of prognosis in patients with sepsis, **(C)** restricted cubic spline models for the relationship between TSH predicted with the risk of prognosis in patients with sepsis, **(D)** restricted cubic spline models for the relationship between FT3/FT4 predicted with the risk of prognosis in patients with sepsis, **(E)** restricted cubic spline models for the relationship between TFQI predicted with the risk of prognosis in patients with sepsis, **(F)** restricted cubic spline models for the relationship between PTFQI predicted with the risk of prognosis in patients with sepsis, **(G)** restricted cubic spline models for the relationship between TSHI predicted with the risk of prognosis in patients with sepsis, **(H)** restricted cubic spline models for the relationship between TT4RI predicted with the risk of prognosis in patients with sepsis.

Restricted cubic spline models revealed nonlinear associations between thyroid hormone levels, sensitivity indices, and sepsis-related mortality (**Figure 1**). Increasing levels of FT3, TFQI, PTFQI, TSHI, and TT4RI were associated with a nonlinear decrease in mortality risk. In contrast, FT4, TSH, and the FT3/FT4 ratio exhibited a U-shaped relationship with mortality, suggesting threshold effects.

### Survival analysis

**Figure 2:**
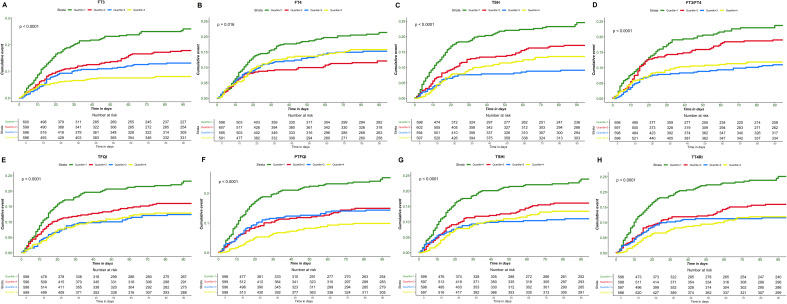
Kaplan-Meier survival curve of sepsis prognosis. **(A)** FT3 different interquartile interval death events, **(B)** FT4 different interquartile interval death events, **(C)** TSH different interquartile interval death events, **(D)** FT3/FT4 different interquartile interval death events, **(E)** TFQI different interquartile interval death events, **(F)** PTFQI different interquartile interval death events, **(G)** TSHI different interquartile interval death events, **(H)** TT4RI different interquartile interval death events.

Kaplan-Meier analysis demonstrated significant differences in 90-day survival by quartiles of thyroid hormone levels and sensitivity indices (**Figure 2**). Patients in the lowest quartiles of FT3, FT4, and TSH exhibited a significantly higher mortality risks compared to those in the highest quartiles (all *p* < 0.05). Similarly, the lowest quartiles of FT3/FT4, TFQI, PTFQI, TT4RI, and TSHI were associated with poorer prognosis compared to the highest quartiles (all *p* < 0.001).

### Cox proportional-hazard regression models

In the unadjusted model (Model 1), all thyroid hormone parameters and sensitivity indices were significant associated with sepsis prognosis (**Supplementary Table S1**; **Table 2**). After fully adjustment for confounders in Model 3, FT3 (HR = 0.95, 95% CI: 0.93–0.98, *p* = 0.001), TSH (HR = 0.89, 95% CI: 0.80–0.99, *p* = 0.004), TFQI (HR = 0.66, 95% CI: 0.51–0.84, p < 0.001), PTFQI (HR = 0.47, 95% CI: 0.37–0.61, *p* < 0.001), TSHI (HR = 0.92, 95% CI: 0.85–0.99, *p* = 0.040), and TT4RI (HR = 0.98, 95% CI: 0.97–0.99, *p* = 0.001) emerged as independent predictors of sepsis prognosis.

**Table 2:** Relationship between thyroid hormone sensitivity index and prognosis of patients with sepsis.

Variables	Model 1		Model 2		Model 3	
	HR (95% CI) a	p value	HR (95% CI) b	p value	HR (95% CI) c	p value
FT3/FT4						
Q1	Reference		Reference		Reference	
Q2	0.74 (0.57-0.97)	0.002	0.78 (0.59-1.01)	0.006	0.85 (0.65-1.12)	0.510
Q3	0.54 (0.40-0.71)	< 0.001	0.62 (0.47-0.83)	< 0.001	0.74 (0.54-1.01)	0.237
Q4	0.47 (0.35-0.63)	< 0.001	0.49 (0.36-0.67)	< 0.001	0.69 (0.51-0.98)	0.054
Continuous variable	0.11 (0.03-0.40)	<0.001	0.20 (0.05-0.74)	0.015	1.19 (0.33-3.90)	0.764
TFQI						
Q1	Reference		Reference		Reference	
Q2	0.72 (0.56-0.94)	0.015	0.71 (0.55-0.93)	0.011	1.09 (0.84-1.40)	0.496
Q3	0.54 (0.41-0.73)	< 0.001	0.56 (0.42-0.75)	< 0.001	0.62 (0.46-0.83)	0.002
Q4	0.53 (0.39-0.70)	< 0.001	0.52 (0.39-0.70)	< 0.001	0.61 (0.46-0.86)	0.003
Continuous variable	0.52 (0.40-0.66)	<0.001	0.53 (0.42-0.68)	<0.001	0.66 (0.51-0.84)	0.001
PTFQI						
Q1	Reference		Reference		Reference	
Q2	0.63 (0.48-0.82)	< 0.001	0.61 (0.47-0.79)	< 0.001	0.60 (0.46-0.78)	< 0.001
Q3	0.59 (0.45-0.77)	< 0.001	0.57 (0.44-0.76)	< 0.001	0.57 (0.44-0.75)	< 0.001
Q4	0.37 (0.28-0.51)	< 0.001	0.39 (0.29-0.53)	< 0.001	0.36 (0.26-0.49)	< 0.001
Continuous variable	0.42 (0.33-0.54)	<0.001	0.45 (0.35-0.57)	<0.001	0.47 (0.37-0.61)	<0.001
TSHI						
Q1	Reference		Reference		Reference	
Q2	0.66 (0.51-0.87)	0.002	0.63 (0.48-0.82)	< 0.001	0.73 (0.56-0.97)	0.030
Q3	0.53 (0.40-0.70)	< 0.001	0.54 (0.41-0.72)	< 0.001	0.66 (0.49-0.89)	0.007
Q4	0.52 (0.39-0.68)	< 0.001	0.52 (0.39-0.69)	< 0.001	0.66 (0.65-1.14)	0.312
Continuous variable	0.81 (0.74-0.88)	<0.001	0.82 (0.76-0.89)	<0.001	0.92 (0.85-0.99)	0.040
TT4RI						
Q1	Reference		Reference		Reference	
Q2	0.62 (0.48-0.81)	< 0.001	0.62 (0.47-0.80)	< 0.001	0.70 (0.53-0.92)	0.010
Q3	0.50 (0.38-0.67)	< 0.001	0.51 (0.38-0.68)	< 0.001	0.89 (0.68-1.16)	0.405
Q4	0.45 (0.33-0.59)	< 0.001	0.45 (0.34-0.61)	< 0.001	0.52 (0.38-0.71)	< 0.001
Continuous variable	0.98 (0.97-0.99)	<0.001	0.98 (0.98-0.99)	<0.001	0.98 (0.97-0.99)	0.001

aModel 1 was a crude model without adjustment for any covariates.

bModel 2 was adjusted for gender, age, marital status, hypertension, coronary heart disease, diabetes, hepatitis, surgery, and mechanical ventilation.

cModel 3 was adjusted for GCS, APACHEII, RBC, WBC, hemoglobin, alanine aminotransferase, serum creatinine, potassium, sodium, chloride, calcium, carbon dioxide, aniongap, glucose, albumin, c-reactive protein and procalcitonin based on Model 2.

### K-means clustering analysis

**Figure 3:**
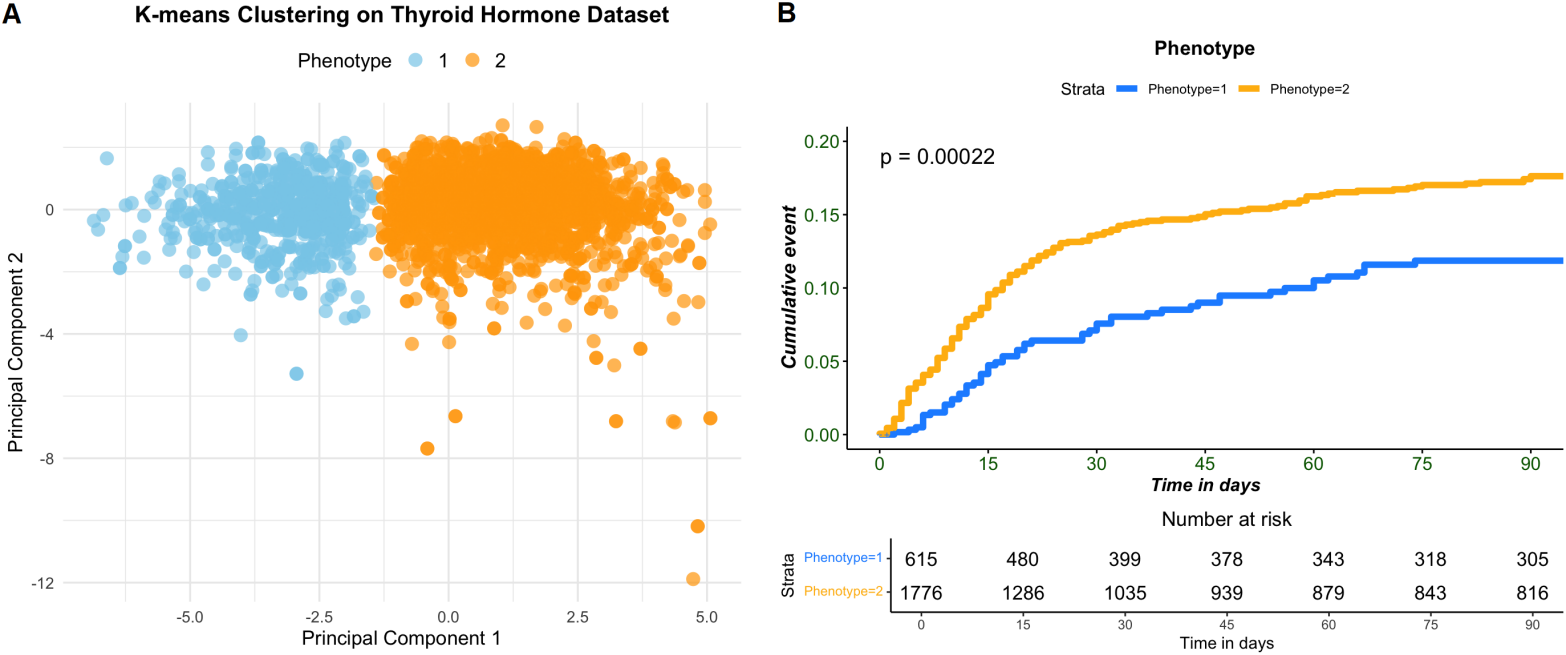
Principal component analysis (PCA) to visualize the clustering results and Kaplan-Meier survival curve. **(A)** After grouping clusters, **(B)** 90-day mortality in different sepsis subphenotypes.

K-means clustering identified two distinct phenotypes based on thyroid hormone profiles (**Supplementary Figure S2**). The two-cluster solution (K = 2) achieved optimal performance, with a Silhouette score of 0.40 and a Calinski-Harabasz score of 1483.16 (**Supplementary Table S2**). Phenotype 1 (n = 615) and Phenotype 2 (n = 1776) differed significantly in baseline thyroid hormone levels and sensitivity indices (**Supplementary Figure S3**). Phenotype 2 exhibited lower FT3, FT4, and TSH levels, as well as reduced FT3/FT4, TFQI, PTFQI, TT4RI, and TSHI values compared to Phenotype 1 (all *p <*0.05). PCA visualized the two phenotypes, with Principal Component 1 and 2 explaining 76.4% of the variance (**Figure 3A**). Kaplan-Meier survival curves demonstrated significantly worse prognosis in Phenotype 2 compared to Phenotype 1 (log-rank *p* < 0.001, **Figure 3B**). The Cox proportional hazards model confirmed that Phenotype 2 was associated with a significantly 36% higher risk of adverse outcomes (HR = 1.42, 95% CI: 1.04–1.91, *p* = 0.029) compared to Phenotype 2 (**Table 3**).

**Table 3:** Relationship between different phenotypes groups and prognosis of sepsis patients (Phenotype 1 as reference).

Models	Hazard ratio (95% CI)	p value
Model 1	1.62 (1.25, 2.10)	< 0.001
Model 2	1.58 (1.21, 2.04)	< 0.001
Model 3	1.42 (1.04, 1.91)	0.029

Model 1 was a crude model without adjustment for any covariates.

Model 2 was adjusted for gender, age, marital status, hypertension, coronary heart disease, diabetes, hepatitis, surgery, and mechanical ventilation.

Model 3 was adjusted for GCS, APACHEII, RBC, WBC, hemoglobin, alanine aminotransferase, serum creatinine, potassium, sodium, chloride, calcium, carbon dioxide, aniongap, glucose, albumin, c-reactive protein and procalcitonin based on Model 2.

## Discussion

This study elucidates the critical role of thyroid hormone sensitivity in sepsis prognosis revealing that impaired thyroid hormone sensitivity, quantified by TFQI, PTFQI, TSHI, and TT4RI, independently predicts 90-day mortality. By integrating central and peripheral thyroid feedback mechanisms, these indices provide a more nuanced assessment of thyroid dysfunction than conventional measurements, offering actionable insights for risk stratification and therapeutic targeting in septic patients.

Different from single thyroid hormone parameters, the composite indices such as TFQI, PTFQI, TSHI, and TT4RI reflect the homeostasis of thyroid hormone more systematically (28). Our findings demonstrate that reduced levels of FT3, FT4, and TSH, alongside with diminished thyroid hormone sensitivity indices, are strongly associated with adverse outcomes in sepsis. Notably, the nonlinear dose-response relationships observed for FT3, TFQI, and PTFQI suggest threshold effects, where even moderate improvements in thyroid sensitivity may confer survival benefits. The identification of two distinct phenotypes—Phenotype 1 (low-risk) and Phenotype 2 (high-risk)—through clustering analysis further underscores the heterogeneity of thyroid dysfunction in sepsis. Phenotype 2, characterized by profound suppression of thyroid hormones and sensitivity indices, exhibited a 36% higher mortality risk, highlighting the potential of thyroid profiles to refine prognostic models. These findings suggest that patients in Phenotype 2 may represent a subset with non-thyroidal illness syndrome (NTIS) or more profound thyroid dysfunction in the context of critical illness. Given their significantly worse prognosis, these patients may benefit from closer monitoring of thyroid function during the ICU stay, particularly in those showing persistent abnormalities.

Thyroid dysfunction during sepsis progression, often termed NTIS, arises from a complex interplay of inflammatory and metabolic derangements (29). Proinflammatory cytokines such as IL-6 and TNF-α disrupt the hypothalamic-pituitary-thyroid (HPT) axis, suppressing the secretion of thyrotropin-releasing hormone (TRH) and TSH, while inhibiting peripheral deiodinase activity, thereby impairing peripheral T4-to-T3 conversion (30, 31). This process is closely associated with “ NTIS”, which is initially an adaptive response to high metabolic demands. However, in sepsis, this response becomes pathological, leading to a state of “tissue hypothyroidism”—where tissue sensitivity to thyroid hormones is significantly reduced despite normal or subnormal circulating hormone levels. Oxidative stress and selenium deficiency further exacerbate deiodinase dysfunction, creating a vicious cycle of reduced T3 production (44). Additionally, inadequate nutritional intake in septic patients suppresses TRH neuronal activity by lowering leptin levels, mimicking the NTIS phenotype observed during starvation, resulting in sustained TSH suppression and reduced thyroid hormone synthesis (32), thereby worsening peripheral metabolic suppression and organ energy crisis.

Reduced thyroid hormone sensitivity worsens sepsis prognosis through multiple pathways. On one hand, T3 deficiency directly impairs mitochondrial function, reducing cellular oxygen utilization and exacerbating metabolic failure in organs such as the heart, liver, and kidneys (33). Low T3 levels have been shown to independently predict the development of chronic critical illness and 28-day mortality in patients with sepsis and septic shock (34). On the other hand, thyroid hormone resistance disrupts immune cell function, weakening innate immune responses and promoting uncontrolled infection and cytokine storms (35). Central sensitivity indices (e.g., reduced TFQI, PTFQI) reflect dysregulation of the HPT axis negative feedback, indicating hypothalamic-pituitary resistance to thyroid hormones, while peripheral sensitivity indices (e.g., reduced FT3/FT4) signify insufficient T3 production. Together, these contribute to a “metabolic deadlock”—where the body cannot upregulate thyroid activity to meet high energy demands. Furthermore, overly sensitive HPT axis feedback may lead to “excessive suppression” of TSH, further limiting thyroid hormone synthesis and creating a vicious cycle (17). Thyroid hormone sensitivity indices (TSHI, TT4RI) quantify the severity of this axis dysregulation by integrating the dynamic relationship between TSH and FT4, thereby more accurately predicting mortality risk and providing targets for early identification of high-risk patients and personalized interventions.

The integration of thyroid hormone sensitivity indices into sepsis care has several clinical implications. Currently, the management of sepsis relies heavily on early recognition, appropriate antimicrobial therapy, and supportive care. However, biomarkers that can predict the severity and outcome of sepsis are limited, and thyroid hormone sensitivity indices may offer an additional tool for clinicians to identify high-risk patients. Patients with high TFQI or low FT3/FT4 ratios may benefit from intensified monitoring and early escalation of therapies. For instance, Phenotype 2 patients might be prioritized for immunomodulatory or organ support interventions. Moreover, while thyroid hormone replacement remains controversial, our findings justify trials of T3/T4 supplementation or thyromimetics in select subgroups (36). Animal studies show that T3 administration improves cardiac output and survival in septic models (37), while human pilot studies suggest potential benefits in NTIS (38). Sensitivity indices could guide patient selection for such interventions.

This study has several limitations. First, its retrospective design precludes causal inferences and introduces the potential for selection bias. Although we adjusted for various covariates, unmeasured confounders (e.g.,selenium levels, and thyroid-related antibody levels) and recall bias may influence the reliability of the results. Second, the single-center design and reliance on a Chinese population may limit the generalizability of our findings. Third, thyroid hormones were measured only at admission, neglecting dynamic changes during ICU stay. Future prospective studies should validate these indices in diverse cohorts and explore their interaction with sepsis phenotypes (e.g., hyperinflammatory vs. immunosuppressive subtypes). Mechanistic research is needed to delineate whether thyroid dysfunction directly exacerbates organ injury or merely serves as a biomarker of disease severity. Furthermore, the utility of these indices in predicting sepsis-related outcomes beyond mortality, such as organ failure or ICU length of stay, warrants investigation. Fourth, inflammatory markers such as IL-6 and TNF-α were not included in our analysis due to substantial missing data, as they are not routinely or systematically measured in clinical practice. Their inclusion in future prospective studies may help clarify the interplay between thyroid function, inflammation, and patient outcomes. Fifth, as this was a retrospective and exploratory study, no predefined clinical cut-off values were established. Although restricted cubic spline analysis suggested potential threshold effects with mortality, prospective studies are needed for validation.

## Conclusion

In conclusion, impaired thyroid hormone sensitivity is an independent prognostic factor in sepsis. These findings position thyroid dysfunction not merely as a bystander but as a modifiable driver of adverse outcomes. By enabling early identification of high-risk patients and informing targeted therapies, thyroid sensitivity indices hold promise for personalizing sepsis management—a critical step toward mitigating its disease burden.

## Data Availability

Publicly available datasets were analyzed in this study. This data can be found here: The data that support the findings of this study are available from the corresponding author upon reasonable request.

## Supplementary material

Supplementary Figure 1 The flowchart of patient selection.

Supplementary Figure 2 The within-cluster variance for the K-means clustering analysis.

Supplementary Figure 3 Selected variables by subphenotype in sepsis and the differences in the standardized values of each variable by subphenotype. All continuous variables were transformed into z-scores.
