# Supplementary figures and images for: Association between sensitivity to thyroid hormone and prognosis in septic patients: a retrospective cohort analysis
Source: Front Endocrinol (Lausanne). 2025 Aug 27;16:1611963. doi: 10.3389/fendo.2025.1611963 (PMC12420208; doi:10.3389/fendo.2025.1611963)

**Supplementary Figure 1.** The flowchart of patient selection.

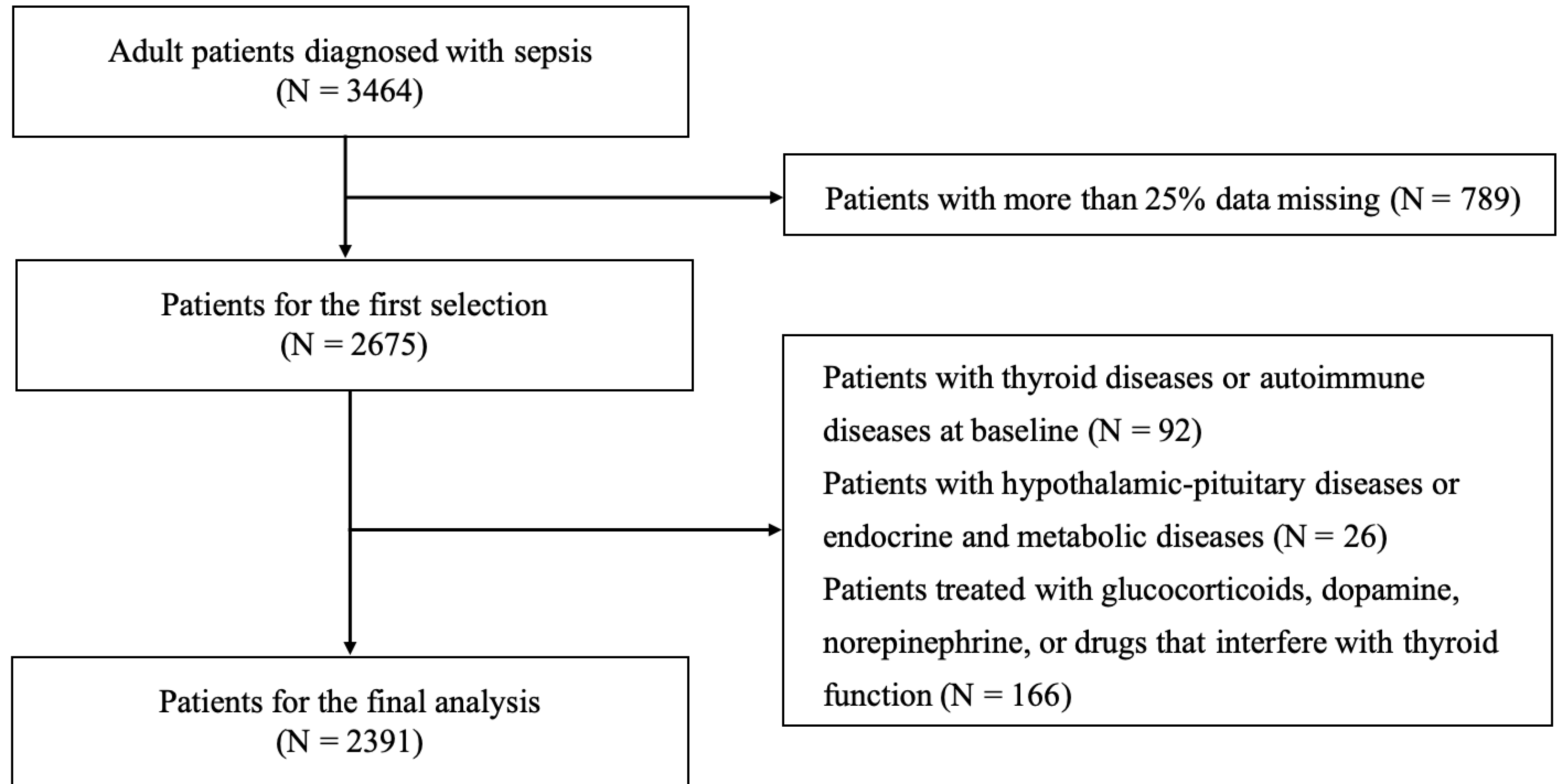

Supplement: Supplementary Figure 1 — The flowchart of patient selection. [file Image1.pdf]

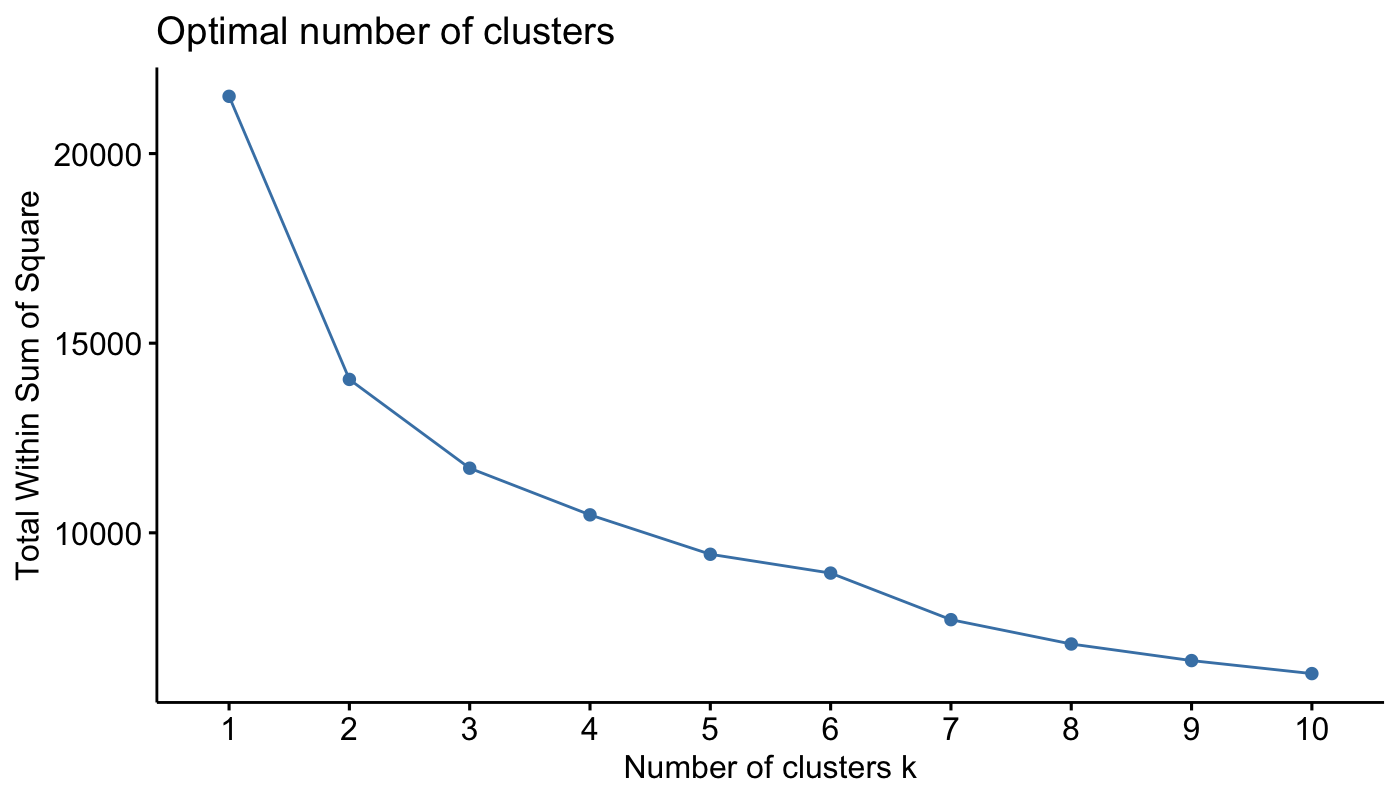

Supplement: Supplementary Figure 2 — The within-cluster variance for the K-means clustering analysis. [file Image2.tiff]

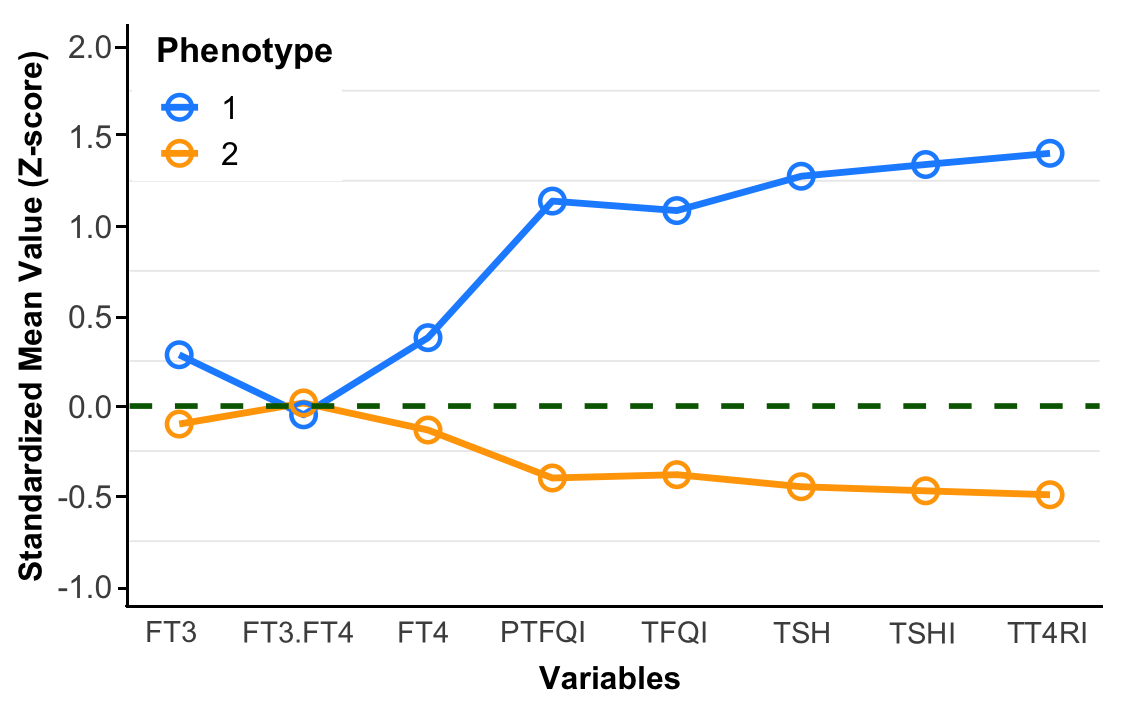

Supplement: Supplementary Figure 3 — Selected variables by subphenotype in sepsis and the differences in the standardized values of each variable by subphenotype. All continuous variables were transformed into z-scores. [file Image3.tiff]
