# Supplementary material for: Association between sensitivity to thyroid hormone and prognosis in septic patients: a retrospective cohort analysis
Source: Front Endocrinol (Lausanne). 2025 Aug 27;16:1611963. doi: 10.3389/fendo.2025.1611963 (PMC12420208; doi:10.3389/fendo.2025.1611963)
Supplement: Supplementary file 4 [file Table1.docx]

**Supplementary Table 1 Relationship between thyroid hormone levels and prognosis in patients with sepsis**

| **Variables** | **Model 1** | | **Model 2** | | **Model 3** | |
| --- | --- | --- | --- | --- | --- | --- |
|  | HR (95% CI) a | p value | HR (95% CI) b | p value | HR (95% CI) c | p value |
| **FT3** |  |  |  |  |  |  |
| Q1 | Reference |  | Reference |  | Reference |  |
| Q2 | 0.62 (0.48-0.81) | < 0.001 | 0.65 (0.50-0.85) | 0.001 | 0.74 (0.56-0.99) | 0.044 |
| Q3 | 0.48 (0.37-0.64) | < 0.001 | 0.55 (0.41-0.73) | < 0.001 | 0.64 (0.48-0.84) | 0.001 |
| Q4 | 0.39 (0.29-0.53) | < 0.001 | 0.46 (0.34-0.63) | < 0.001 | 0.59 (0.44-0.78) | < 0.001 |
| Continuous variable (pmol/L) | 0.73 (0.67-0.81) | < 0.001 | 0.76 (0.69-0.84) | < 0.001 | 0.95 (0.93-0.98) | 0.001 |
| **FT4** |  |  |  |  |  |  |
| Q1 | Reference |  | Reference |  | Reference |  |
| Q2 | 0.76 (0.58-0.99) | 0.048 | 0.76 (0.57-0.99) | 0.048 | 0.86 (0.66-1.11) | 0.255 |
| Q3 | 0.74 (0.56-0.97) | 0.034 | 0.74 (0.56-0.98) | 0.039 | 0.95 (0.71-1.28) | 0.774 |
| Q4 | 0.65 (0.49-0.86) | 0.002 | 0.64 (0.48-0.85) | 0.002 | 0.74 (0.54-1.01) | 0.063 |
| Continuous variable (pmol/L) | 0.97 (0.95-1.00) | 0.069 | 0.97 (0.95-1.00) | 0.068 | 0.91 (0.82-1.00) | 0.071 |
| **TSH** |  |  |  |  |  |  |
| Q1 | Reference |  | Reference |  | Reference |  |
| Q2 | 0.70 (0.54-0.91) | 0.007 | 0.70 (0.54-0.90) | 0.006 | 0.75 (0.57-0.98) | 0.036 |
| Q3 | 0.53 (0.41-0.70) | < 0.001 | 0.55 (0.42-0.73) | < 0.001 | 0.46 (0.33-0.63) | < 0.001 |
| Q4 | 0.41 (0.31-0.56) | < 0.001 | 0.40 (0.30-0.55) | < 0.001 | 0.76 (0.58-1.00) | 0.051 |
| Continuous variable (mIU/L) | 0.81 (0.74-0.89) | < 0.001 | 0.82 (0.75-0.90) | <0.001 | 0.89 (0.80-0.99) | 0.004 |

a Model 1 was a crude model without adjustment for any covariates.

b Model 2 was adjusted for gender, age, marital status, hypertension, coronary heart disease, diabetes, hepatitis, surgery, and mechanical ventilation.

c Model 3 was adjusted for GCS, APACHE Ⅱ, RBC, WBC, hemoglobin, alanine aminotransferase, serum creatinine, potassium, sodium, chloride, calcium, carbon dioxide, aniongap, glucose, albumin, c-reactive protein and procalcitonin based on Model 2.
