# Supplementary material for: Association between sensitivity to thyroid hormone and prognosis in septic patients: a retrospective cohort analysis
Source: Front Endocrinol (Lausanne). 2025 Aug 27;16:1611963. doi: 10.3389/fendo.2025.1611963 (PMC12420208; doi:10.3389/fendo.2025.1611963)
Supplement: Supplementary file 5 [file Table2.docx]

**Supplementary Table 2. Fit statistics for the K-means clustering analysis from two to nine classes.**

| **Class number** | **Silhoutte score** | **Davies-Bouldin score** | **Calinski-Harabasz score** |
| --- | --- | --- | --- |
| 2 | 0.402 | 1.075 | 1483.157 |
| 3 | 0.328 | 1.625 | 844.023 |
| 4 | 0.277 | 1.467 | 997.635 |
| 5 | 0.233 | 1.544 | 856.894 |
| 6 | 0.242 | 1.519 | 737.150 |
| 7 | 0.241 | 1.508 | 799.635 |
| 8 | 0.236 | 1.512 | 740.863 |
| 9 | 0.240 | 1.395 | 724.510 |

**Silhouette score**, a measure of how similar a sample is to its own cluster, ranged from -1 to 1—values closer to 1 being better.

**Davies–Bouldin Score**, a measure of the average similarity of each cluster with its most similar cluster, ranged from zero upward—values closer to zero being better.

**Calinski–Harabasz score**, the ratio between the within-cluster dispersion and the between-cluster dispersion, ranged from zero upward—higher values being better for a dataset.
